# Atractylodin alleviates neuroinflammation and protects neurological function after ischemic stroke in association with reduced NLRP3 inflammasome activation

**DOI:** 10.3389/fneur.2026.1754614

**Published:** 2026-03-27

**Authors:** Qingyao Ouyang, Yiying Yang, Hoi Leong Lee, Ziyu Yan, Dezhi Deng, Wei Li

**Affiliations:** 1 College of Basic Medical Sciences, China Three Gorges University, Yichang, Hubei, China; 2 Nanyang Second General Hospital, Nanyang, Henan, China; 3 Biomedical Electronic Engineering Programme, Faculty of Electronic Engineering and Technology, Universiti Malaysia Perlis, Arau, Perlis, Malaysia; 4 Department of Neurology, The Affiliated Hospital of Jiaxing University, Jiaxing, China; 5 Department of Clinical Laboratory, The Affiliated Hospital of Jiaxing University, Jiaxing, China

**Keywords:** atractylodin, ischemic stroke, neuroinflammation, neuroprotection, NLRP3 inflammasome

## Abstract

**Background:**

NOD-like receptor protein 3 (NLRP3) inflammasome–driven neuroinflammation contributes to ischemic stroke injury. Atractylodin (ART) shows anti-inflammatory activity, but its neuroprotective potential and mechanistic links to NLRP3 signaling after cerebral ischemia–reperfusion (I/R) injury remain to be defined.

**Materials and methods:**

BV2 microglia were subjected to oxygen–glucose deprivation/reoxygenation (OGD/R), and rats underwent middle cerebral artery occlusion (MCAO) followed by reperfusion. ART was administered after reperfusion *in vivo* (10 or 30 mg/kg, once daily for 3 days) and during reoxygenation *in vitro*. Neurobehavioral outcomes and infarct volume were assessed in MCAO rats, along with measurements of neuronal apoptosis. NLRP3 inflammasome activation, gasdermin D (GSDMD)–dependent pyroptosis, pro-inflammatory cytokines, and microglial polarization markers were evaluated using immunoblotting, immunofluorescence, RT-qPCR, and enzyme-linked immunosorbent assay (ELISA).

**Results:**

In OGD/R-stimulated BV2 microglia, ART suppressed NLRP3 inflammasome activation and reduced GSDMD cleavage, accompanied by decreased IL-1β and IL-18 production. In the MCAO model, ART significantly improved neurological outcomes and reduced infarct volume and neuronal apoptosis. These protective effects were linked to a reduction in the expression of NLRP3 pathway components (NLRP3, ASC, and caspase-1) and GSDMD-N. Additionally, there was a shift in microglial responses toward an anti-inflammatory (M2-like) profile, which led to a decrease in pro-inflammatory markers.

**Conclusion:**

ART confers neuroprotection in experimental ischemic stroke by inhibiting NLRP3 inflammasome–associated pyroptosis and modulating microglial inflammatory polarization, supporting its potential as a therapeutic candidate for ischemic stroke.

## Introduction

Ischemic stroke is a clinical syndrome caused by cerebral neuronal ischemia and represents the most prevalent form of cerebrovascular disease worldwide, posing a significant threat to human health (1). Current clinical strategies primarily focus on restoring cerebral blood flow through thrombolytic therapy or surgical thrombectomy (2). Although these approaches can be effective in selected patients, many individuals continue to experience significant neurological deficits, leading to long-term disability and imposing significant emotional and economic burdens on patients and their families (3). Previous research indicates that the pathogenesis of ischemic stroke involves oxidative stress, blood–brain barrier (BBB) disruption, and inflammatory responses (4). Among these mechanisms, ischemia-induced neuroinflammation plays a key role in ischemic brain injury and represents one of the most common forms of secondary brain damage following stroke (5, 6). Therefore, regulating post-ischemic neuroinflammation is considered a promising therapeutic strategy for improving stroke prognosis.

Microglia are the resident immune cells of the central nervous system (CNS) and are essential for immune surveillance, initiation of inflammation, and clearance of cellular debris. Following cerebral ischemia, microglia become rapidly activated and initiate inflammatory cascades (7). Activated microglia are generally classified into two phenotypes: pro-inflammatory (M1-like) microglial state and anti-inflammatory/reparative (M2-like) microglial state (8). M1-polarized microglia promote tissue injury by releasing pro-inflammatory mediators, including tumor necrosis factor-α (TNF-α), interleukin-1β (IL-1β), and platelet-derived growth factor (PDGF), exacerbating inflammation, disrupting the BBB, damaging neurons, inducing apoptosis, and impairing neuroprotection (9, 10). M2-polarized microglia express markers, such as arginase-1 (Arg-1) and interleukin-10 (IL-10), and secrete insulin-like growth factor-1 (IGF-1), which support angiogenesis, tissue repair, and inflammation resolution (11, 12). Therefore, shifting microglial polarization toward the anti-inflammatory/reparative (M2-like) microglial state may attenuate neuroinflammation and improve neurological function after ischemic stroke.

The NOD-like receptor protein 3 (NLRP3) inflammasome is a cytosolic multiprotein complex composed of the sensor NLRP3, the adaptor apoptosis-associated speck-like protein containing a caspase recruitment domain (ASC), and the effector pro-caspase-1 (13). The activation of this complex leads to caspase-1 cleavage, which facilitates the maturation and secretion of IL-1β and IL-18, thereby increasing neuroinflammatory responses (14). Furthermore, activated caspase-1 cleaves gasdermin D (GSDMD), triggering pyroptosis—a distinct form of programmed cell death characterized by the release of inflammatory mediators (15, 16). NLRP3 inflammasomes have been identified as key contributors to both acute injury and secondary brain damage following ischemic stroke (17). Pharmacological inhibition of NLRP3, including the selective inhibitor MCC950, has been shown to reduce neurological deficits in ischemic injury (18, 19). However, the precise mechanisms through which NLRP3 inflammasomes mediate ischemia-induced brain injury remain incompletely understood, highlighting the therapeutic potential of targeting NLRP3-associated neuroinflammation.

Atractylodin (ART) is the principal active compound isolated from the rhizomes of *Atractylodes lancea*. It is classified as a polyyne and is known for its anti-inflammatory, antioxidant, antitumor, and gastroprotective properties (20). Recent studies have reported that ART alleviates neuroinflammation and neuronal injury in models of brain inflammation and depression (21), suppresses lipopolysaccharide-induced microglial activation and inflammatory responses (22), and attenuates acute lung injury, which is linked to a reduction in NLRP3 inflammasome activation (23). Despite these results, the potential protective role of ART in ischemic stroke has not been fully explored. Therefore, this study employs a rat ischemia–reperfusion (I/R) model and an oxygen–glucose deprivation/reoxygenation (OGD/R) microglial model to investigate the neuroprotective and anti-neuroinflammatory effects of ART in ischemic stroke.

## Materials and methods

### Materials and reagents

**Figure 1:**
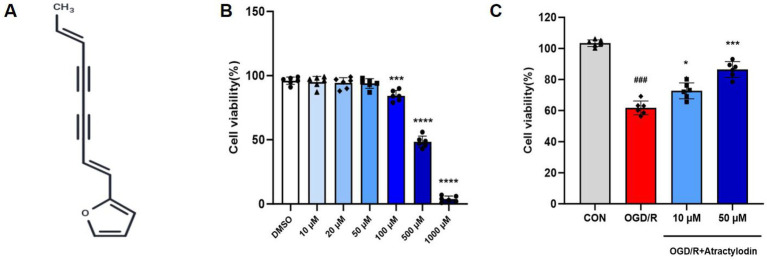
Effects of atractylodin (ART) on microglial cell viability. **(A)** Chemical structure of ART. **(B)** BV2 microglial cells were seeded into 96-well plates, cultured overnight, and treated with the indicated concentrations of ART (10, 20, 50, 100, 500, and 1,000 μM) for 24 h. **(C)** BV2 cells were exposed to OGD/R and treated with atractylodin (10 and 50 μM). Cell viability was assessed using the CCK-8 assay and expressed as a percentage of the control group. Data are presented as mean ± SEM (*n* = 6). ***p < 0.001, ****p < 0.0001 vs. DMSO; ^###^*p* < 0.001 vs. control group; *p < 0.05, **p < 0.01, ***p < 0.001 vs. OGD/R group.

Atractylodin (ART; CAS No. 55290-63-6) was purchased from TopScience Biotechnology Co., Ltd. (Shanghai, China) and its chemical structure is shown in Figure 1. Antibodies against NLRP3, caspase-1, and GSDMD-N were obtained from Thermo Fisher Scientific (USA), while antibodies against mature IL-1β and interleukin-18 (IL-18) were purchased from Abcam (USA). Fetal bovine serum (FBS) and Dulbecco’s Modified Eagle’s Medium (DMEM) were obtained from Thermo Fisher Scientific. RNA extraction kits were purchased from Tiangen Biotech Co., Ltd. (Beijing, China). Enzyme-linked immunosorbent assay (ELISA) kits and the Cell Counting Kit-8 (CCK-8) were obtained from Boyan Biotech Co., Ltd. (Nanjing, China).

### Cell culture

BV2 microglial cells were cultured in DMEM supplemented with 10% FBS and 1% penicillin–streptomycin in a humidified incubator at 37 °C with 5% CO_2_ and 20% O_2_. The cells were passaged, and the culture medium was refreshed every 2 days. For experiments, the cells were seeded 24 h before treatment and cultured in glucose-free DMEM. To establish the OGD/R model, the cells were placed in a hypoxic chamber containing 95% N_2_ and 5% CO_2_ at 37 °C for 2 h, followed by reoxygenation in normal DMEM under normoxic conditions (20% O_2_, 5% CO_2_) for 24 h. ART treatment was initiated at the onset of reoxygenation and continued throughout the 24-h reoxygenation phase.

### Cell viability assessment

Cell viability was assessed using the CCK-8 assay. BV2 cells were treated with various concentrations of ART (0, 10, 20, 50, 100, 500, and 1,000 μM) for 24 h. Subsequently, the CCK-8 reagent was added following the manufacturer’s instructions, and the cells were incubated for an additional 3 h. Absorbance was measured at 450 nm using a microplate reader.

### Middle cerebral artery occlusion (MCAO) model

Healthy male Sprague–Dawley (SD) rats (250 ± 30 g) were obtained from the Experimental Animal Center of Three Gorges University (Yichang, Hubei Province, China). All experimental procedures were approved by the Animal Ethics Committee of Three Gorges University (Approval No. 2023070Z4). A total of 48 rats were randomly assigned to four groups (*n* = 12 per group): (1) Sham group, in which the left common carotid artery was exposed without occlusion; (2) MCAO group, in which rats underwent left middle cerebral artery occlusion using the Longa filament method for 1.5 h, followed by reperfusion; (3) MCAO + ART (10 mg/kg) group, in which rats received oral ART at a dose of 10 mg/kg once daily for 3 consecutive days after reperfusion; and (4) MCAO + ART (30 mg/kg) group, in which rats received oral ART at a dose of 30 mg/kg once daily for 3 consecutive days after reperfusion. The first dose was administered immediately after reperfusion. ART was administered immediately after reperfusion to target early inflammatory activation during the acute phase of ischemia–reperfusion (I/R) injury. The once-daily dosing for 3 consecutive days was chosen to target the early post-ischemic inflammatory period.

### Neurological function scoring

Neurological deficits were evaluated using the modified neurological severity score (mNSS) at 0 h, 24 h, 2 d, and 3 d after reperfusion. The mNSS assesses motor, sensory, reflex, and balance functions, with scores ranging from 0 to 18. Higher scores indicate more severe neurological impairment. A score of 1–6 indicates mild neurological impairment, 7–12 indicates moderate impairment, and 13–18 indicates severe neurological dysfunction.

### 2,3,5-triphenyl-tetrazolium chloride (TTC) staining

At 72 h after reperfusion (day 3), rats were anesthetized and euthanized for TTC staining and subsequent tissue collection. The rats were deeply anesthetized with isoflurane and transcardially perfused with ice-cold normal saline. The brains were rapidly removed and sectioned into five coronal slices using a rodent brain matrix. Slices were incubated in 2% 2,3,5-triphenyltetrazolium chloride (TTC) solution at 37 °C for 30 min in the dark. Images were captured digitally, and infarct volumes were quantified using ImageJ software (NIH). Infarct volume was calculated as the ratio of infarcted tissue to total brain volume.

### Hematoxylin and eosin (H&E) staining

The brain tissues from the ischemic penumbra were fixed, dehydrated, paraffin-embedded, and sectioned coronally at a thickness of 5 μm. Sections were dried at 60 °C for 4 h, deparaffinized in xylene, and rehydrated through a graded ethanol series. After hematoxylin and eosin (H&E) staining, the sections were dehydrated, cleared in xylene, mounted with neutral resin, and examined under a light microscope.

### Nissl staining

Ischemic penumbral brain tissues from each group were frozen and coronally sectioned at a thickness of 10 μm using a cryostat at −20 °C. The sections were air-dried, stained with Nissl solution for 5 min, differentiated in 95% ethanol, cleared in xylene, and mounted with neutral resin. Nissl-positive neurons were then observed and imaged using a light microscope.

### TUNEL staining

Terminal deoxynucleotidyl transferase dUTP nick end labeling (TUNEL) staining was performed using a commercial kit (Roche, Germany) following the manufacturer’s instructions to detect apoptotic cells in brain tissue. After transcardial perfusion, the brains were fixed in 4% paraformaldehyde overnight, paraffin-embedded, and sectioned coronally at a thickness of 5 μm. The sections were deparaffinized and rehydrated, followed by incubation with proteinase K (20 μg/mL) at room temperature for 15 min. Endogenous peroxidase activity was quenched using 3% hydrogen peroxide. They were then incubated with the TUNEL reaction mixture at 37 °C for 1 h in a humidified chamber and counterstained with DAPI. Images were acquired using a fluorescence microscope. The number of TUNEL-positive nuclei and total DAPI-stained nuclei was counted in five randomly selected high-power fields (400×) per section. The apoptosis index was calculated as the ratio of TUNEL-positive cells to total nuclei.

### Western blotting

After treatment, the rats were transcardially perfused with ice-cold phosphate-buffered saline (PBS). Ischemic cortical tissues (approximately 20 mg) were homogenized in radioimmunoprecipitation assay (RIPA) buffer supplemented with protease inhibitors. Homogenates were centrifuged at 12,000 × g for 20 min at 4 °C, and protein concentrations in the supernatants were determined using a bicinchoninic acid (BCA) assay. Equal amounts of protein (20–40 μg) were separated by SDS–PAGE and transferred onto polyvinylidene fluoride (PVDF) membranes. Membranes were blocked with 5% skim milk overnight and incubated at 4 °C with primary antibodies against NLRP3, pro-caspase-1 (45 kDa), cleaved caspase-1 (p20), ASC, GSDMD-N, mature IL-1*β*(p17), IL-18, and β-actin (1:1,000). After washing with Tris-buffered saline containing 0.1% Tween-20 (TBST), membranes were incubated with horseradish peroxidase-conjugated secondary antibodies (1:10,000) at room temperature for 2 h. Protein bands were visualized using enhanced chemiluminescence (ECL) reagents and quantified using ImageJ software.

### Immunofluorescence staining

Immunofluorescence staining was performed to assess target protein expression in ischemic brain tissue. The rats were transcardially perfused, and the brains were fixed overnight at 4 °C, followed by cryoprotection in 30% sucrose. Tissues were embedded in optimal cutting temperature (OCT) compound and sectioned at a thickness of 20–30 μm. The sections were washed with PBS, permeabilized with 0.3% Triton X-100, and blocked with 5% bovine serum albumin (BSA) for 1 h at room temperature. They were then incubated overnight at 4 °C with primary antibodies against IBA1 and NLRP3. After PBS washes, they were incubated with fluorescent secondary antibodies for 1 h at room temperature and counterstained with DAPI. Images were captured using fluorescence and confocal microscopy, and quantitative analysis was performed using ImageJ.

### RT-qPCR analysis

Total RNA was extracted from ischemic cortical tissues using the TRIzol reagent (Invitrogen, CA, USA). RNA purity and concentration were assessed using a NanoDrop spectrophotometer, with acceptable A260/A280 ratios of 1.8–2.0. Complementary DNA (cDNA) was synthesized using a reverse transcription kit (Novoprotein, Suzhou, China). Quantitative real-time PCR was performed using SYBR Green Master Mix (Novoprotein) on a real-time PCR system following the manufacturer’s instructions. Target genes included *NLRP3, ASC, caspase-1, CD16, CD32*, inducible nitric oxide synthase (*iNOS*), *CD206*, arginase-1 (*Arg1*), and *CCL22*. *GAPDH* served as the internal reference gene. Relative mRNA expression levels were calculated using the 2^−ΔΔCt^ method, and all reactions were performed in triplicate. Primer sequences are listed in Table 1.

**Table 1:** Primers for RT-qPCR.

Gene	Forward primer sequence (5′–3′)	Reverse primer sequence (5′–3′)
NLRP3	ATGCTGCTTCGACATCTCCT	AACAATGCGAGATCCTGAC
Caspase-1	AAACGCCATGGCTGACAAGA	GTCCCGTGCCTTGTCCATAG
ASC	TTGCTGGATGCTCTGTATGG	AGGCTGGAGCAAAGCTAAA
CD16	AGGAGGAGACCATCTTGTGG	TGTAGATGAGGAGGGGCTGT
CD32	CTGGAGGAGCTGATGATGAG	ACATGGTGAGGAAGAGAGC
iNOS	CAGCTGGGCTGTACAAACCTT	CATTGGAAGTGAAGCGTTTCG
CD206	GGCTCTGTTCAGCTATTGGAC	GATGGCATTGGTGATTCAGGG
Arg1	CTCCAAGCCAAAGTCCTTAGAG	AGGAGCTGTCATTAGGGACATC
CCL22	GTGCTGCTCCTGGTTCTC	TGAGGTCACTTGTGTGCTG
GAPDH	GAAGGTGAAGGTCGGAGTC	GAAGATGGTGATGGGATTTC

### ELISA detection

Levels of IL-1β (Kit No. EK0394) and IL-18 (Kit No. EK0736) in ischemic brain tissues were measured using commercial ELISA kits (Boyan Biotechnology Co., Ltd., China) following the manufacturer’s instructions. The brain tissues were homogenized in ice-cold saline and centrifuged to obtain supernatants. Aliquots (100 μL) were added to ELISA plates and incubated at 37 °C for 2 h. After washing, enzyme conjugates and chromogenic substrates were sequentially added, followed by incubation in the dark for 15 min. The reaction was terminated using a stop solution, and absorbance was measured with a microplate reader. Cytokine concentrations were calculated based on standard curves and expressed as picograms per milligram of protein (pg/mg).

### Randomized grouping and blind method

The animals were randomly allocated to four groups (*n* = 12/group). Neurological function (mNSS) was assessed in all animals, while infarct volume and histology were analyzed in a randomly selected subset (*n* = 6/group). Molecular assays (WB, RT-qPCR, ELISA, and IF) were performed on additional randomly selected tissues (*n* = 3/group). Outcome assessments were conducted in a blinded manner. Exclusion criteria included SAH, failed occlusion, and intraoperative death.

### Statistical analysis

Data are presented as mean ± standard error of the mean (SEM). Statistical analyses were performed using GraphPad Prism 9.0 (GraphPad Software, La Jolla, CA, USA). Comparisons among multiple groups were performed using one-way analysis of variance (ANOVA), followed by Tukey’s *post-hoc* test. Comparisons between two groups were performed using an unpaired Student’s *t*-test. A *p*-value of < 0.05 was considered statistically significant.

## Results

### Effect of ART on microglial viability

The cytotoxic effects of ART were evaluated in BV2 microglial cells using the CCK-8 assay (Figure 1A). Cells were treated with increasing concentrations of ART (0, 10, 20, 50, 100, 500, and 1,000 μM) for 24 h, and cell viability was assessed. As shown in Figure 1B, ART at concentrations up to 50 μM did not significantly reduce cell viability, whereas exposure to 100 μM resulted in a significant decrease in viability, indicating concentration-dependent cytotoxicity. Based on these results, 10 μM was selected as a non-cytotoxic concentration, and 50 μM was chosen as a higher, near-cytotoxic concentration for subsequent *in vitro* experiments. To further determine whether ART alleviates OGD/R-induced BV2 cell death, the CCK-8 assay was performed following OGD/R stimulation (Figure 1C). OGD/R markedly reduced cell viability compared to the control group. ART treatment significantly restored cell viability in a dose-dependent manner, with the 50 μM group exhibiting a more pronounced protective effect.

### Neuroprotective effects of ART in MCAO rats

**Figure 2:**
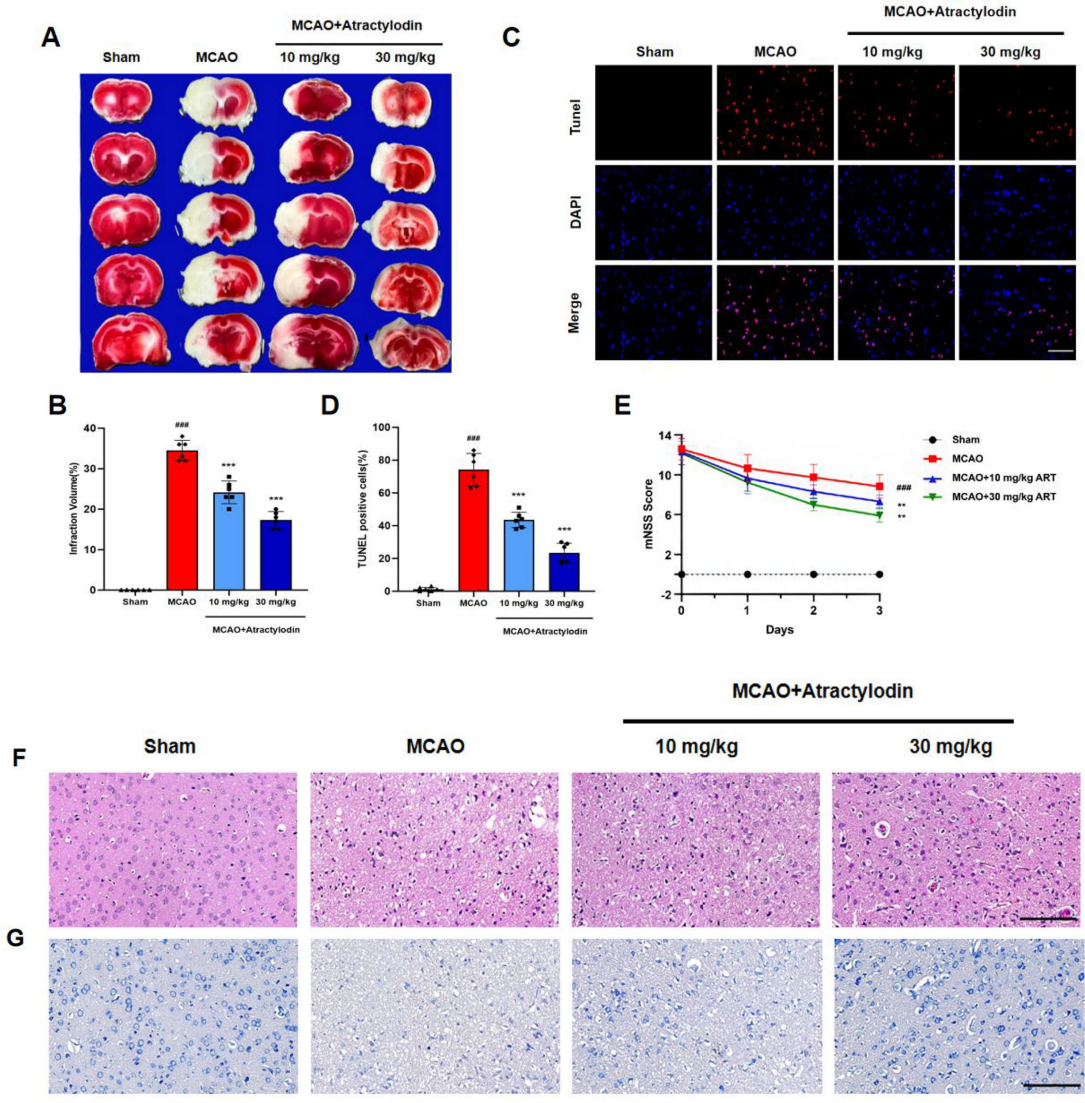
Neuroprotective effects of atractylodin on cerebral ischemia–reperfusion injury in rats. **(A)** Representative TTC-stained brain sections. **(B)** Quantification of cerebral infarct volume (*n* = 6). **(C)** Representative TUNEL staining images showing neuronal apoptosis (*n* = 6). **(D)** Quantitative analysis of neuronal apoptosis, expressed as the percentage of TUNEL-positive cells (*n* = 6). **(E)** The modified neurological severity score (mNSS) evaluated over three consecutive days following ischemia–reperfusion injury (*n* = 12). **(F)** Representative hematoxylin and eosin (H&E)-stained sections. **(G)** Representative Nissl-stained sections. Data are presented as mean ± SEM. ^###^*p* < 0.001 vs. sham group; **p < 0.01, ***p < 0.001 vs. MCAO group. Scale bar = 100 m.

To investigate the neuroprotective effects of ART in cerebral ischemia, a MCAO rat model was established. Neurological function, cerebral infarct volume, neuronal apoptosis, and histopathological changes were evaluated. TTC staining revealed no infarction in the sham group, whereas a significant infarct area was observed in the MCAO group, confirming the successful establishment of the model. Treatment with ART significantly reduced infarct volume in a dose-dependent manner compared to the MCAO group (Figures 2A,B). Neuronal apoptosis was assessed by TUNEL staining. The MCAO group showed a significant increase in TUNEL-positive cells compared to the sham group, while ART treatment significantly reduced the proportion of apoptotic neurons (Figures 2C,D). Neurological function was assessed using the mNSS. Rats subjected to MCAO displayed significantly elevated mNSS scores, indicating severe neurological impairment. ART treatment significantly decreased mNSS scores, with the higher dose (30 mg/kg) producing a more pronounced improvement in neurological function (Figure 2E).

Histopathological analysis further supported these results. H&E and Nissl staining demonstrated intact neuronal morphology and orderly cellular arrangement in the sham group. The MCAO group showed disrupted neuronal architecture, cell shrinkage, nuclear condensation, and significant loss of Nissl substance. ART treatment alleviated these pathological changes, as evidenced by improved neuronal organization, reduced neuronal necrosis, and preservation of Nissl bodies (Figures 2F,G). These results indicate that ART reduces ischemia–reperfusion-induced brain injury and preserves neurological function.

### ART’S effect on microglia inflammation and polarization in MCAO rats

**Figure 3:**
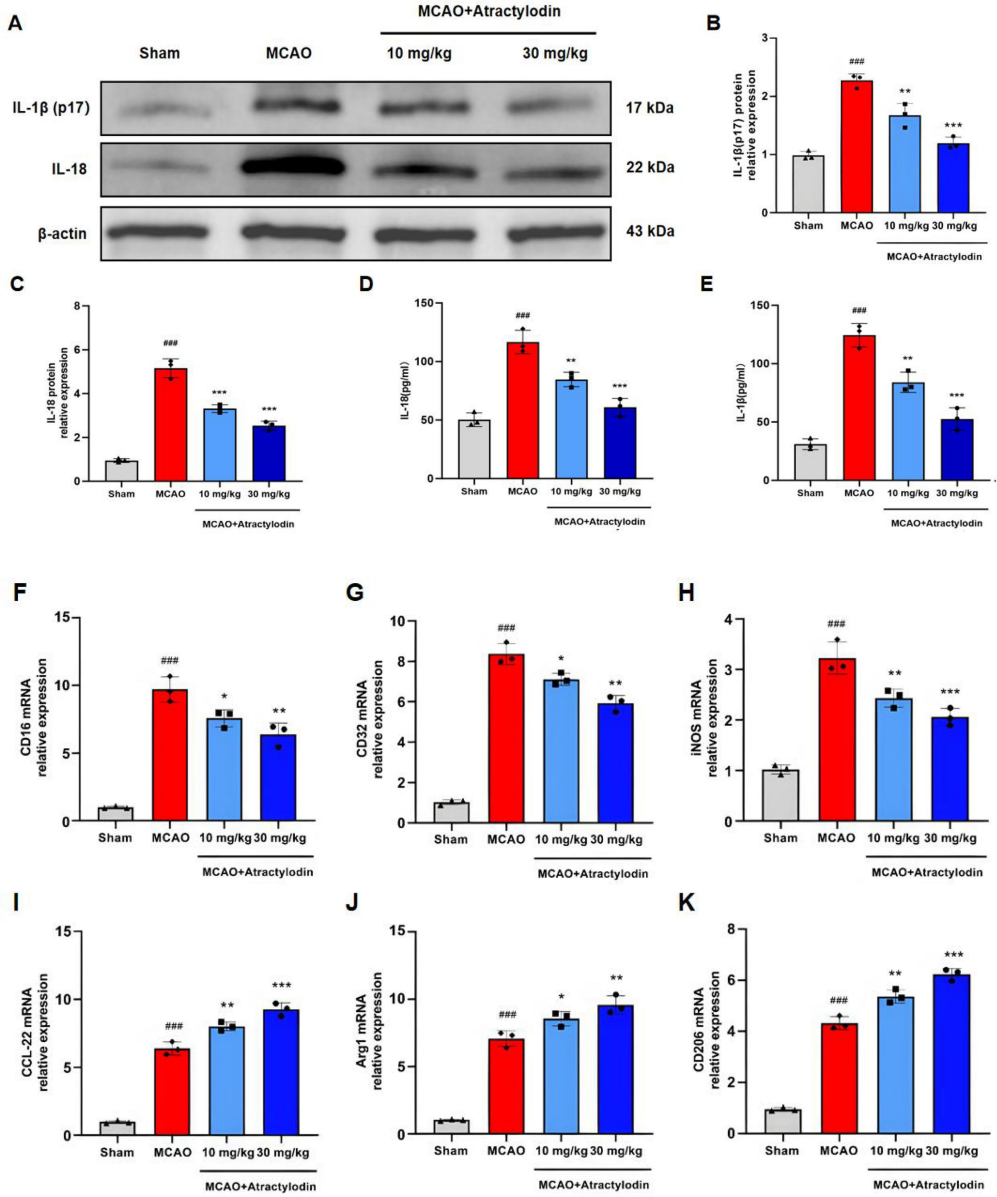
Atractylodin suppresses the inflammatory response and promotes an anti-inflammatory/reparative microglial activation bias in MCAO rats. **(A)** Representative western blot images showing mature IL-1β (p17) and IL-18 protein expression. **(B,C)** Densitometric analysis of mature IL-1β (p17) and IL-18 protein levels. **(D,E)** ELISA measurements of IL-1β and IL-18 levels in brain tissue homogenates. **(F–H)** RT-qPCR analysis of M1 microglial marker mRNA expression (CD16, CD32, and iNOS). **(I–K)** RT-qPCR analysis of M2 microglial marker mRNA expression (CD206, Arg1, and CCL-22). Data are presented as mean ± SEM (*n* = 3 per group). ^###^*p* < 0.001 vs. sham group; *p < 0.05, **p < 0.01, ***p < 0.001 vs. MCAO group.

To elucidate the immunomodulatory mechanisms underlying the neuroprotective effects of ART, microglial inflammatory responses and polarization were examined in ischemic brain tissue. Western blot analysis showed that the protein levels of the pro-inflammatory cytokines mature IL-1β (p17) and IL-18 were significantly elevated in the MCAO group compared to the sham group (Figures 3A–C). ART treatment significantly reduced the expression of mature IL-1β (p17) and IL-18 in a dose-dependent manner. Consistent results were obtained using ELISA, confirming that ART administration decreased the levels of mature IL-1β (p17) and IL-18 (Figures 3D,E). Microglial polarization was further assessed by RT-qPCR analysis of M1- and M2-associated markers. The mRNA levels of M1 markers (CD16, CD32, and iNOS) and M2 markers (CD206, Arg1, and CCL22) were significantly elevated in the MCAO group relative to the sham group, indicating microglial activation after ischemic injury (Figures 3F–K). ART treatment significantly downregulated M1-associated gene expression while promoting M2-associated gene expression in a dose-dependent manner. These findings indicate that ART suppresses microglia-mediated inflammatory responses and promotes a shift toward an anti-inflammatory microglial phenotype following cerebral ischemia.

### ART mitigates dendritic neurons’ pyroptosis after cerebral I/R injury

**Figure 4:**
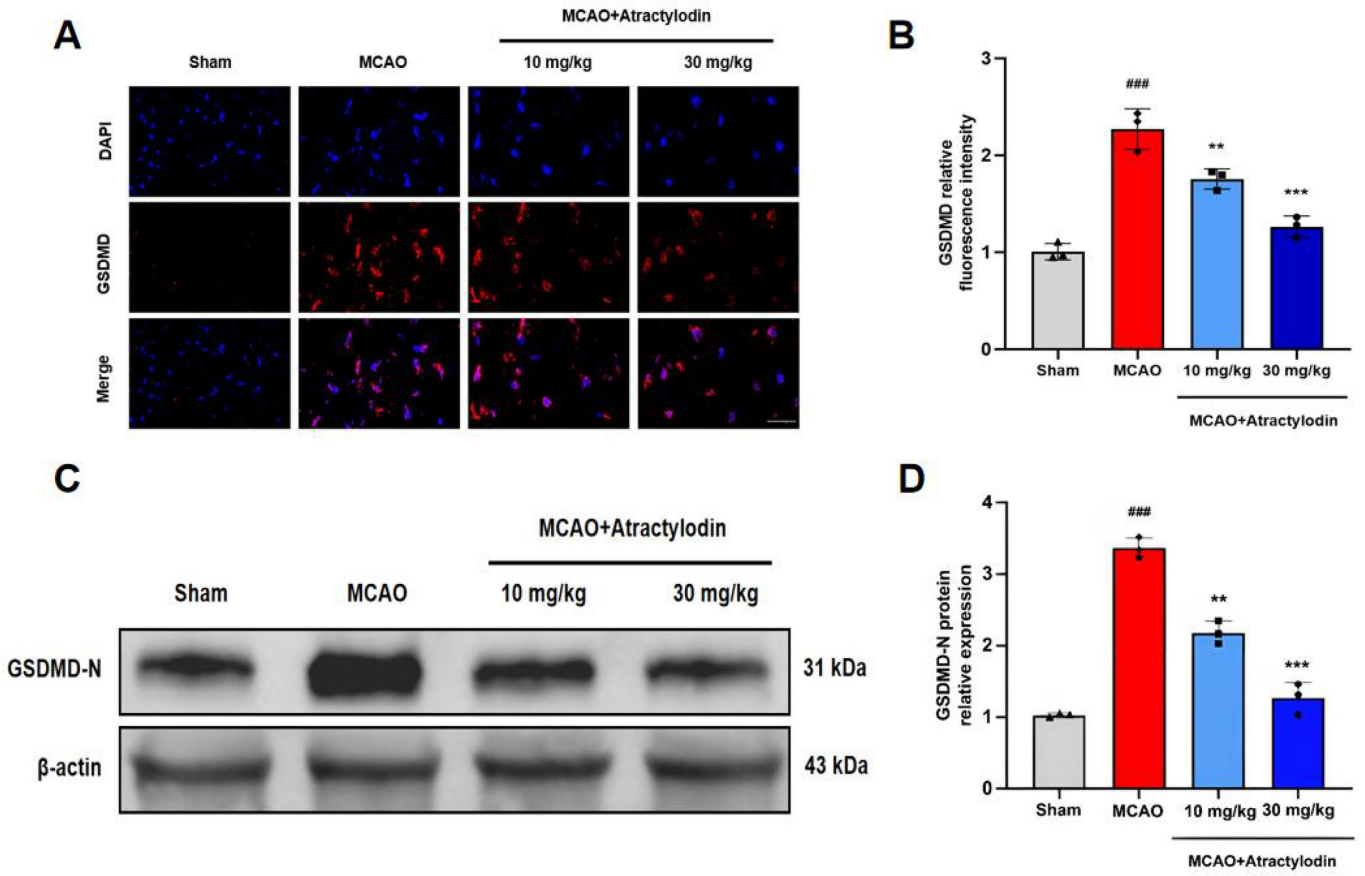
Atractylodin reduces GSDMD-N expression in ischemic brain regions. **(A)** Representative immunofluorescence images showing GSDMD staining (red) in brain sections. **(B)** Quantification of GSDMD immunofluorescence intensity. **(C)** Representative western blot images showing GSDMD-N protein expression. **(D)** Densitometric analysis of GSDMD-N protein levels normalized to β-actin, with control values set to 100%. Data are presented as mean ± SEM (*n* = 3). ^###^*p* < 0.001 vs. sham group; **p < 0.01, ***p < 0.001 vs. MCAO group. Scale bar = 50 μm.

To investigate whether ART modulates pyroptosis following cerebral I/R injury, GSDMD expression was evaluated. Immunofluorescence analysis revealed a significant increase in GSDMD fluorescence intensity in the ischemic regions of MCAO rat brains compared to sham controls. ART treatment significantly reduced GSDMD fluorescence intensity (Figures 4A,B). To further confirm the inhibitory effect of ART on pyroptosis, we detected the N-terminal fragment of GSDMD (GSDMD-N), the active executor of pyroptotic cell death. Western blot analysis showed that GSDMD-N expression was markedly increased after cerebral I/R injury, whereas ART treatment significantly reduced GSDMD-N levels in a dose-dependent manner (Figures 4C,D).

### ART suppresses NLRP3 Inflammasome activity after cerebral I/R injury

**Figure 5:**
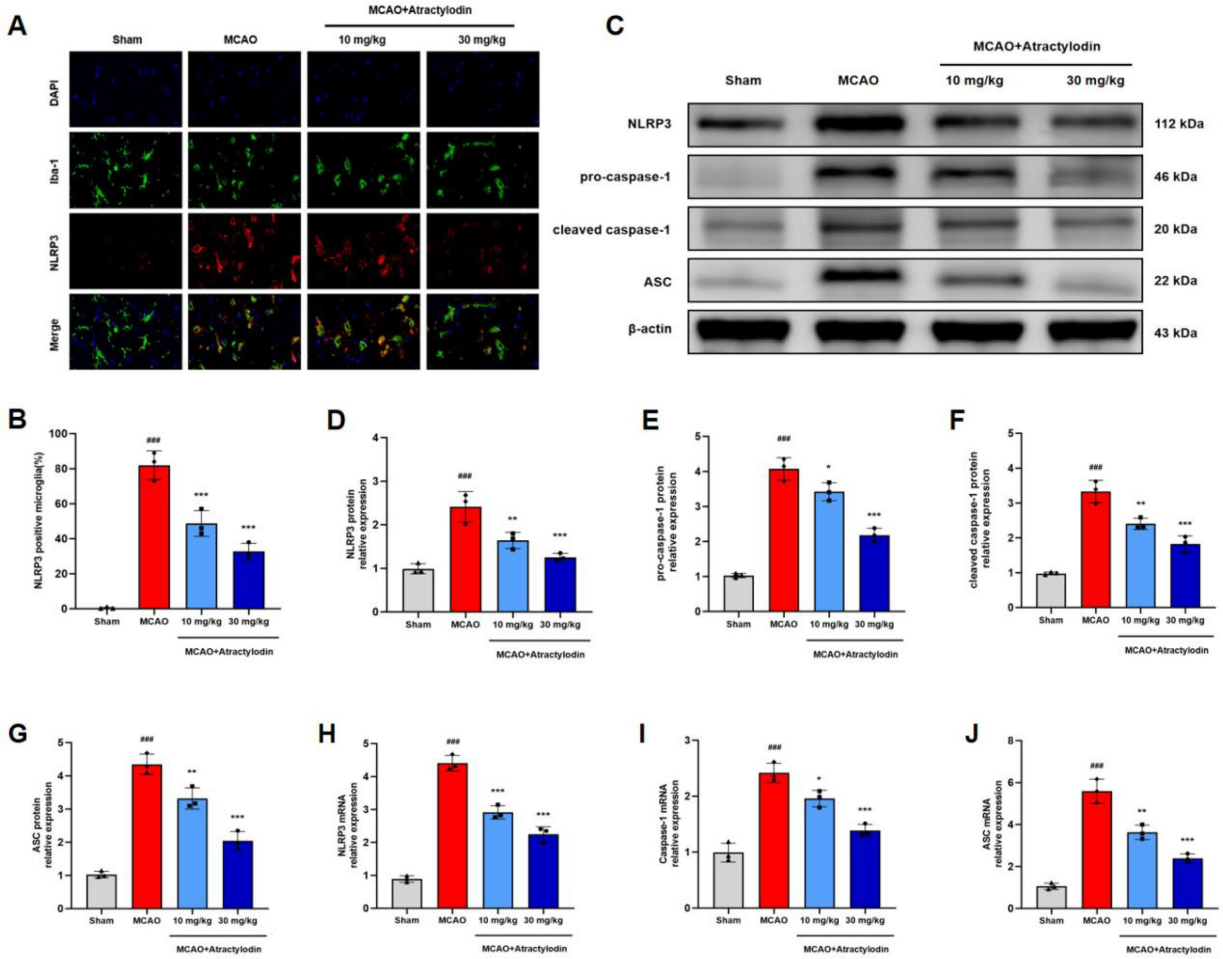
Atractylodin suppresses NLRP3 inflammasome activation following cerebral ischemia–reperfusion injury. **(A)** Representative immunofluorescence images showing co-localization of NLRP3 (red) and Iba1 (green) in brain tissue. **(B)** Quantification of NLRP3-positive microglial cells. **(C)** Representative western blot images showing NLRP3, ASC, pro-caspase-1 (45 kDa), and cleaved caspase-1 (p20) protein expression. **(D–G)** Densitometric analysis of protein levels normalized to *β*-actin. **(H–J)** RT-qPCR analysis of NLRP3, ASC, and caspase-1 mRNA expression. Data are presented as mean ± SEM (*n* = 3). ^###^*p* < 0.001 vs. sham group; **p < 0.01, ***p < 0.001 vs. MCAO group. Scale bar = 100 m.

To further elucidate the anti-neuroinflammatory and anti-pyroptotic mechanisms of ART, the activation of the NLRP3 inflammasome was examined in ischemic brain tissue. Immunofluorescence staining revealed a significant increase in NLRP3-positive microglia in the ischemic hemisphere of MCAO rats compared to sham controls. This increase was significantly reduced by ART treatment (Figures 5A,B). Western blot analysis showed that the protein levels of NLRP3, ASC, pro-caspase-1 (45 kDa), and cleaved caspase-1 (p20) were significantly elevated in the MCAO group, indicating the activation of the NLRP3 inflammasome. ART treatment reduced the expression of NLRP3, ASC, pro-caspase-1, and cleaved caspase-1 (p20) in a dose-dependent manner (Figures 5C–G). Similarly, RT-qPCR analysis demonstrated that ART significantly downregulated the mRNA levels of NLRP3, ASC, and caspase-1 following I/R injury (Figures 5H–J). These results indicate that ART is associated with reduced NLRP3 inflammasome activation after cerebral reperfusion.

### Effects of ART on BV2 microglial inflammation and polarization after OGD/R

**Figure 6:**
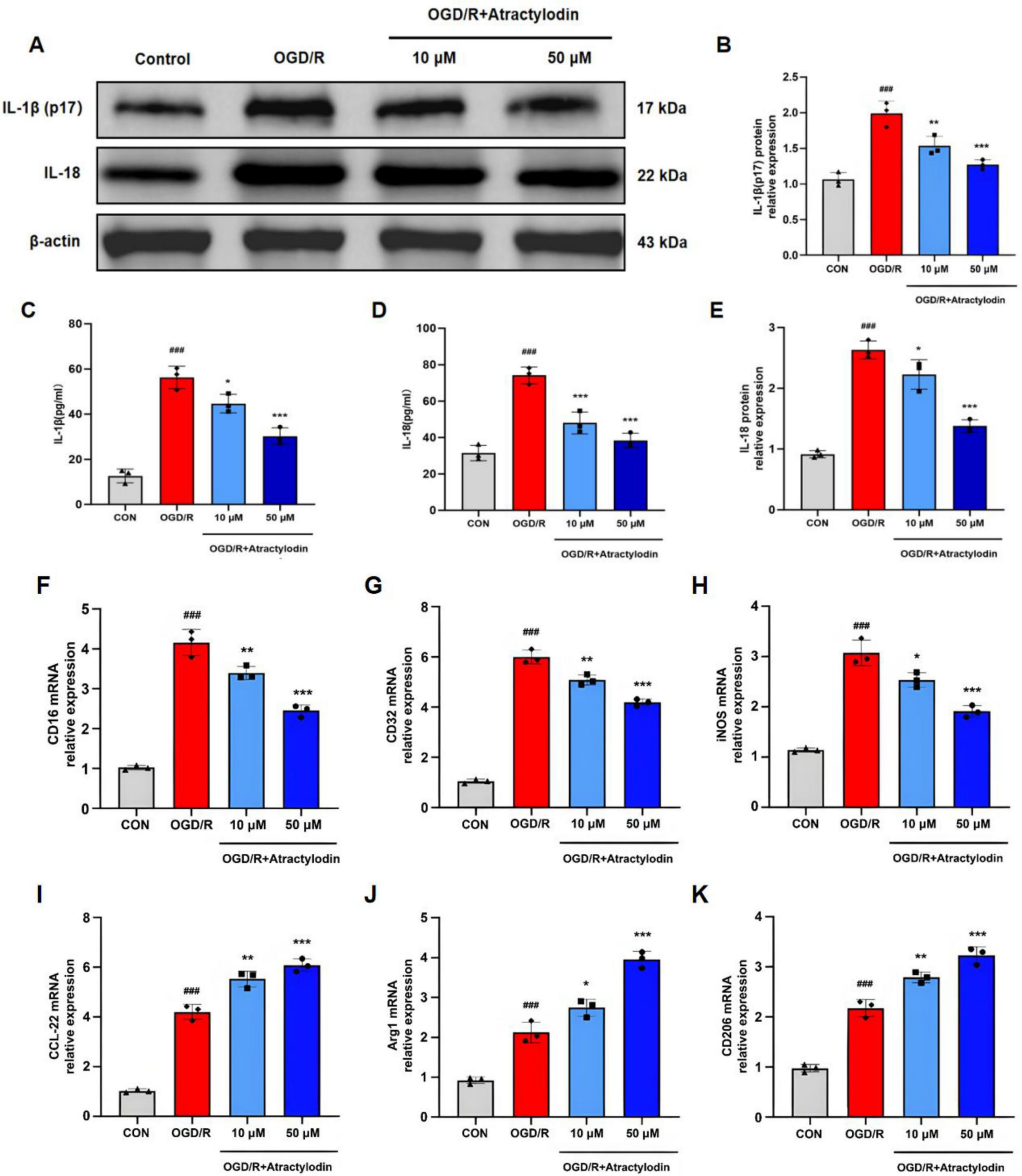
Atractylodin attenuates inflammation and promotes an anti-inflammatory/reparative microglial activation bias in BV2 microglia following OGD/R stimulation. **(A)** Representative western blot images showing mature IL-1β (p17) and IL-18 levels in BV2 cell supernatants. **(B,C)** Densitometric analysis of mature IL-1β (p17) and IL-18 protein levels. **(D,E)** ELISA quantification of IL-1β and IL-18 levels in BV2 cell supernatants. **(F–K)** RT-qPCR analysis of M1 markers (CD16, CD32, and iNOS) and M2 markers (CD206, Arg1, and CCL-22) in BV2 cells. Data are presented as mean ± SEM (*n* = 3). ^###^*p* < 0.001 vs. control group; *p < 0.05, **p < 0.01, ***p < 0.001 vs. OGD/R group.

To confirm the anti-inflammatory and immunomodulatory effects of ART at the cellular level, BV2 microglia subjected to OGD/R were analyzed. Western blot analysis showed that OGD/R stimulation significantly increased the protein levels of mature IL-1β (p17) and IL-18 in BV2 cells. Treatment with ART at concentrations of 10 and 50 μM significantly reduced the expression of mature IL-1β (p17) and IL-18 in a dose-dependent manner (Figures 6A–C). Treatment with ART at concentrations of 10 and 50 µM significantly reduced mature IL-1β (p17) and IL-18 expression in a dose-dependent manner (Figures 6D,E). Microglial polarization was further assessed by RT-qPCR. OGD/R stimulation significantly increased the expression of M1-associated genes (CD16, CD32, and iNOS) and moderately increased the expression of M2-associated genes (CD206, Arg1, and CCL22). ART treatment significantly suppressed the expression of M1 markers while promoting the expression of M2 markers in a dose-dependent manner (Figures 6F–K). These findings indicate that ART attenuates inflammatory responses in OGD/R-stimulated microglia and promotes a shift toward an anti-inflammatory marker-defined profile phenotype. The *in vitro* results are consistent with the *in vivo* observations.

### ART attenuates pyroptosis in BV2 microglia following OGD/R

**Figure 7:**
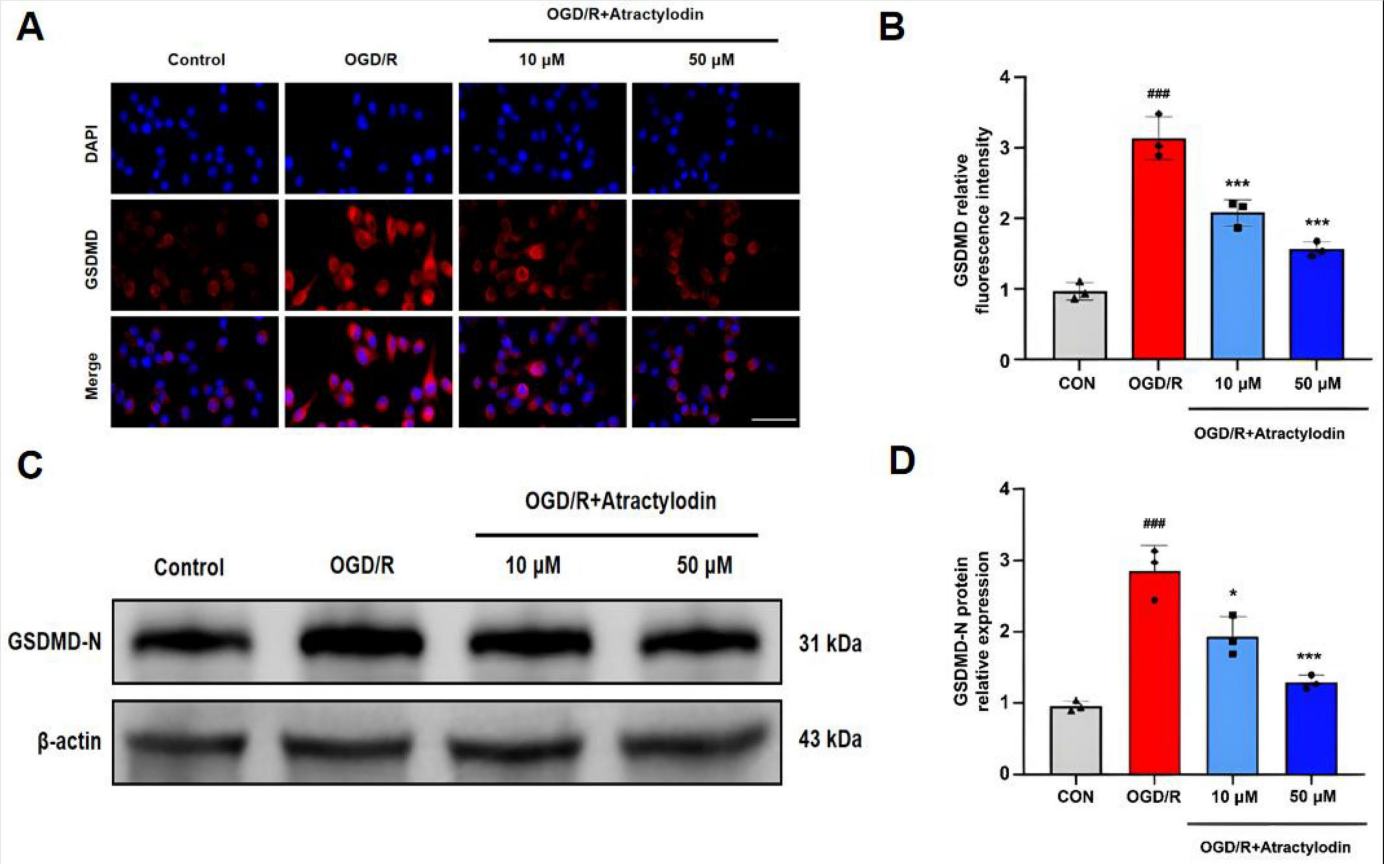
Atractylodin reduces GSDMD-N expression in BV2 microglia after OGD/R. **(A)** Representative immunofluorescence images showing GSDMD staining (red) in BV2 microglia. **(B)** Quantification of GSDMD immunofluorescence intensity. **(C)** Representative western blot images showing GSDMD-N protein expression in BV2 cell lysates. **(D)** Densitometric analysis of GSDMD-N protein levels normalized to β-actin. Data are presented as mean ± SEM (*n* = 3). ^###^*p* < 0.001 vs. control group; **p < 0.01, ***p < 0.001 vs. OGD/R group. Scale bar = 50 μm.

To determine whether ART regulates pyroptosis in microglia after OGD/R, GSDMD expression was examined. Immunofluorescence staining showed that OGD/R significantly increased GSDMD fluorescence intensity in BV2 microglia, whereas ART treatment significantly reduced GSDMD expression in a concentration-dependent manner (Figures 7A,B). To further confirm the inhibitory effect of ART on pyroptosis in BV2 microglia following OGD/R, we examined the N-terminal fragment of gasdermin D (GSDMD-N), the active executor of pyroptotic cell death. Western blot analysis revealed that OGD/R stimulation markedly increased GSDMD-N expression in BV2 microglia, whereas ART treatment significantly reduced GSDMD-N levels in a dose-dependent manner (Figures 7C,D). These results indicate that ART inhibits OGD/R-induced pyroptosis in BV2 microglia.

### ART inhibits NLRP3 inflammasome activation in BV2 microglia after OGD/R

**Figure 8:**
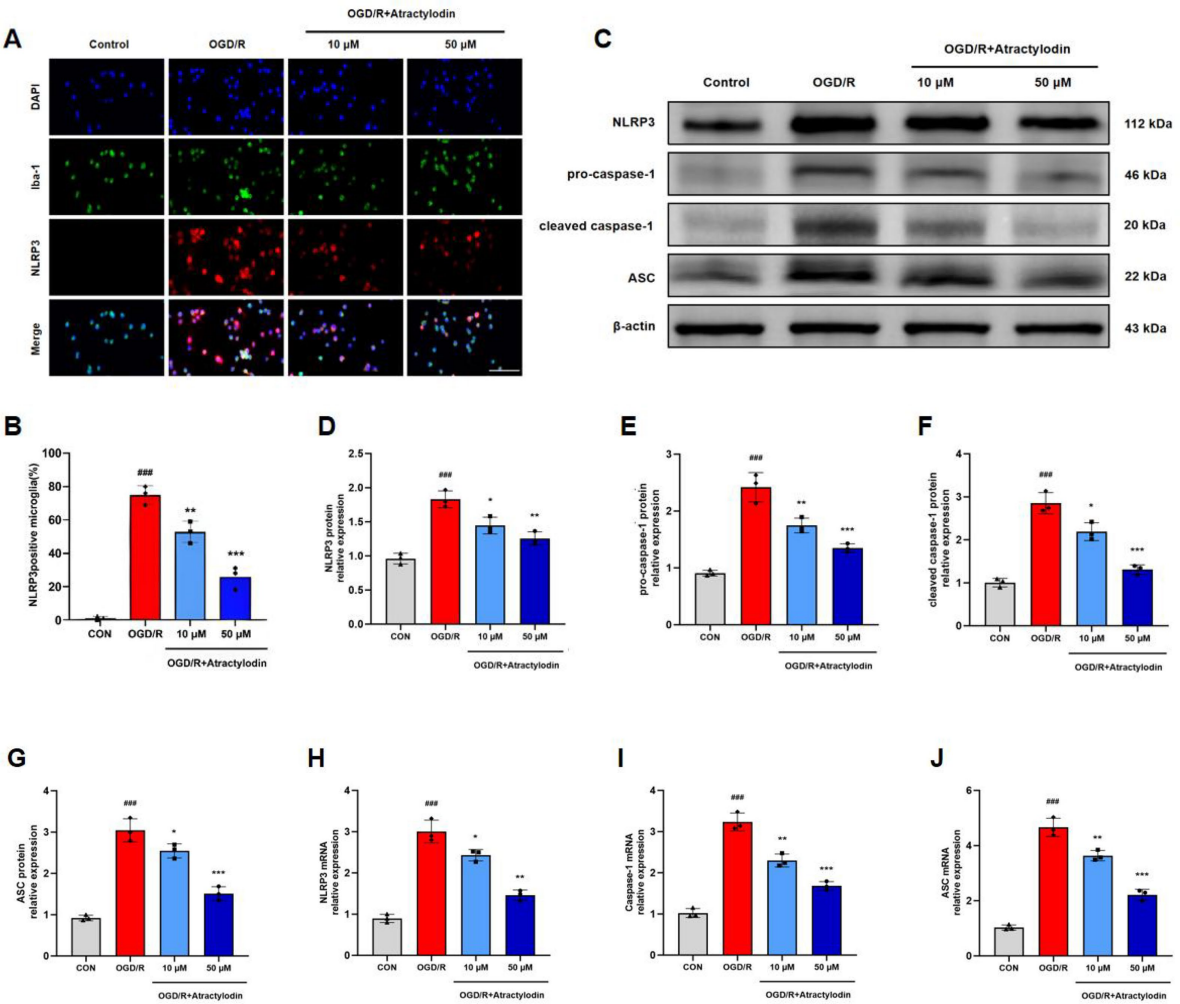
Atractylodin inhibits NLRP3 inflammasome activation in BV2 microglia after OGD/R. **(A)** Representative immunofluorescence images and quantification of Iba1 and NLRP3 double-positive BV2 microglia. **(B)** Quantification of NLRP3-positive BV2 cells. **(C)** Representative western blot images showing NLRP3, ASC, pro-caspase-1 (45 kDa), and cleaved caspase-1 (p20) protein expression, with β-actin as the loading control. **(D–G)** Densitometric analysis of NLRP3, ASC, and cleaved caspase-1 protein levels. **(H–J)** RT-qPCR analysis of NLRP3, ASC, and caspase-1 mRNA expression in BV2 microglia, normalized to a housekeeping gene. Data are presented as mean ± SEM (*n* = 3). ^###^*p* < 0.001 vs. control group; *p < 0.05, **p < 0.01, ***p < 0.001 vs. OGD/R group. Scale bar = 100 μm.

The effects of ART on NLRP3 inflammasome activation in BV2 microglia were further examined. Immunofluorescence analysis showed that OGD/R stimulation significantly increased the number of NLRP3-positive BV2 cells, whereas ART treatment reduced NLRP3 expression in a dose-dependent manner (Figures 8A,B). Western blot analysis demonstrated that OGD/R significantly increased the protein levels of NLRP3, ASC, pro-caspase-1 (45 kDa), and cleaved caspase-1 (p20). ART treatment markedly reduced the expression of cleaved caspase-1 (p20), indicating suppression of inflammasome activation (Figures 8C–G). Moreover, RT-qPCR analysis showed that ART reduced the mRNA levels of NLRP3, ASC, and caspase-1 in a dose-dependent manner in OGD/R-treated BV2 microglia (Figures 8H–J). These results demonstrate that ART is associated with reduced NLRP3 inflammasome activation in microglia following OGD/R in a concentration-dependent manner.

## Discussion

Neuroinflammation and secondary injury are major contributors to neurological dysfunction following ischemic stroke. Among the cellular mediators involved, microglia play a central role by dynamically responding to ischemic stress and shaping the post-ischemic inflammatory microenvironment. Excessive or prolonged activation of pro-inflammatory microglial responses amplifies neuronal injury, whereas a shift toward anti-inflammatory and reparative microglial functions facilitates tissue repair and functional recovery. Therefore, therapeutic strategies that rebalance microglial inflammatory responses are considered promising for improving stroke outcomes.

In the present study, ART treatment shifted post-ischemic microglial responses toward a neuroprotective profile. Rather than simply suppressing inflammation, ART appeared to modulate the balance between detrimental and reparative microglial activities. This immunomodulatory effect is particularly relevant, as sustained pro-inflammatory signaling after ischemia has been shown to exacerbate blood–brain barrier disruption, neuronal apoptosis, and neurological deficits. By promoting an anti-inflammatory microenvironment, ART may create conditions that support neuronal survival and structural preservation after ischemia–reperfusion injury.

The observed neuroprotective effects of ART were closely associated with the inhibition of NLRP3 inflammasome signaling. Activation of the NLRP3 inflammasome is a key driver of post-ischemic neuroinflammation, linking ischemic stress to caspase-1 activation, the maturation of IL-1β and IL-18, and gasdermin D–mediated pyroptotic cell death. This cascade establishes a feed-forward inflammatory loop that amplifies tissue damage. Our findings suggest that ART interrupts this pathological signaling axis, thereby limiting inflammasome-driven cytokine release and pyroptosis. The consistency between *in vivo* and *in vitro* observations supports a central role for NLRP3 inflammasome suppression in ART-mediated neuroprotection.

Previous studies have reported anti-inflammatory and antioxidant properties of ART in peripheral inflammatory disorders and neuroinflammatory conditions unrelated to ischemic stroke. The present study extends these findings by providing evidence that ART confers protection in experimental cerebral ischemia and links its effects to coordinated regulation of microglial inflammatory polarization and inflammasome-associated pyroptosis. This integrated mechanism distinguishes ART from agents that target single inflammatory mediators and highlights its potential as a multi-target therapeutic candidate for ischemic stroke.

Several limitations should be acknowledged. First, although our data demonstrate a strong association between ART treatment and suppression of NLRP3 inflammasome signaling, causal relationships were not directly assessed. Future studies using genetic or pharmacological inhibition of NLRP3, such as MCC950 or knockout models, will be necessary to confirm mechanistic specificity. Second, long-term functional recovery beyond the acute phase was not evaluated and therefore warrants further investigation. The therapeutic window and optimal dosing regimen of ART were not systematically examined and therefore require further investigation. Third, although BV2 cells are widely used for mechanistic studies, primary microglia may better reflect physiological conditions *in vivo*. Future studies will incorporate primary microglial cultures to further validate the pharmacological effects of ART. In addition, we acknowledge that some molecular and histological analyses were performed with relatively small sample sizes, which may limit statistical robustness. Therefore, the findings should be interpreted with appropriate caution, and further studies with larger sample sizes are needed to validate these results. Although ART cytotoxicity was evaluated, cell viability under OGD/R conditions was not directly quantified using the CCK-8 or LDH assay, which requires further investigation. Further study of upstream signaling pathways regulating NLRP3 activation is necessary to fully elucidate the molecular mechanisms of ART.

## Conclusion

This study demonstrates that ART alleviates cerebral I/R injury by inhibiting neuroinflammation, promoting microglial polarization toward the anti-inflammatory/reparative (M2-like) microglial state, inhibiting NLRP3 inflammasome activation, and reducing pyroptosis. These findings provide experimental evidence supporting ART as a potential therapeutic candidate for the treatment of ischemic stroke.

## Data Availability

The datasets presented in this study can be found in online repositories. The names of the repository/repositories and accession number(s) can be found in the article/Supplementary material.

## References

[1] GBD 2019 Stroke Collaborators. Global, regional, and national burden of stroke and its risk factors, 1990-2019: a systematic analysis for the global burden of disease study 2019. Lancet Neurol. (2021) 20:795–820. doi: 10.1016/S1474-4422(21)00252-0, 34487721 PMC8443449

[2] Krishnamurthi RV Krishnamurthi R V Ikeda T Feigin V L. Global, regional and country-specific burden of ischaemic stroke, intracerebral haemorrhage and subarachnoid haemorrhage: a systematic analysis of the global burden of disease study 2017. Neuroepidemiology. (2020) 54:171–9. doi: 10.1159/000506396, 32079017

[3] Kuriakose D Xiao Z. Pathophysiology and treatment of stroke: present status and future perspectives. Int J Mol Sci. (2020) 21:7609. doi: 10.3390/ijms21207609, 33076218 PMC7589849

[4] Chamorro Á Dirnagl U Urra X Planas AM. Neuroprotection in acute stroke: targeting excitotoxicity, oxidative and nitrosative stress, and inflammation. Lancet Neurol. (2016) 15:869–81. doi: 10.1016/S1474-4422(16)00114-9, 27180033

[5] Zietz A Gorey S Kelly PJ Katan M McCabe JJ. Targeting inflammation to reduce recurrent stroke. Int J Stroke. (2024) 19:379–87. doi: 10.1177/17474930231207777, 37800305 PMC10964390

[6] Lian L Zhang Y Liu L Yang L Cai Y Zhang J . Neuroinflammation in ischemic stroke: focus on MicroRNA-mediated polarization of microglia. Front Mol Neurosci. (2021) 13:612439. doi: 10.3389/fnmol.2020.612439, 33488360 PMC7817943

[7] He J Liu J Huang Y Tang X Xiao H Hu Z. Oxidative stress, inflammation, and autophagy: potential targets of mesenchymal stem cells-based therapies in ischemic stroke. Front Neurosci. (2021) 15:641157. doi: 10.3389/fnins.2021.641157, 33716657 PMC7952613

[8] Ransohoff RM. A polarizing question: do M1 and M2 microglia exist? Nat Neurosci. (2016) 19:987–91. doi: 10.1038/nn.4338, 27459405

[9] Jiang CT Wu WF Deng YH Ge JW. Modulators of microglia activation and polarisation in ischemic stroke (review). Mol Med Rep. (2020) 21:2006–18. doi: 10.3892/mmr.2020.11003, 32323760 PMC7115206

[10] Wang G Li X Li N Wang X He S Li W . Icariin alleviates uveitis by targeting peroxiredoxin 3 to modulate retinal microglia M1/M2 phenotypic polarisation. Redox Biol. (2022) 52:102297. doi: 10.1016/j.redox.2022.102297, 35334248 PMC8956882

[11] Li X Shi H Zhang D Jing B Chen Z Zheng Y . Paeonol alleviates neuropathic pain by modulating microglial M1 and M2 polarization via the RhoA/p38MAPK signaling pathway. CNS Neurosci Ther. (2023) 29:2666–79. doi: 10.1111/cns.14211, 37032648 PMC10401133

[12] Yang X Xu S Qian Y Xiao Q. Resveratrol regulates microglia microglial inflammatory activation profile via PGC-1α in conditions of neuroinflammatory injury. Brain Behav Immun. (2017) 64:162–72. doi: 10.1016/j.bbi.2017.03.003, 28268115

[13] Fu J Wu H. Structural mechanisms of NLRP3 inflammasome assembly and activation. Annu Rev Immunol. (2023) 41:301–16. doi: 10.1146/annurev-immunol-081022-021207, 36750315 PMC10159982

[14] Qin Z Shi D-D Li W Cheng D Zhang Y-D Zhang S . Berberine ameliorates depression-like behaviours in mice via inhibiting NLRP3 inflammasome-mediated neuroinflammation and preventing neuroplasticity disruption. J Neuroinflammation. (2023) 20:54. doi: 10.1186/s12974-023-02744-7, 36859349 PMC9976521

[15] Paik S Kim JK Silwal P Sasakawa C Jo E-K. An update on the regulatory mechanisms of NLRP3 inflammasome activation. Cell Mol Immunol. (2021) 18:1141–60. doi: 10.1038/s41423-021-00670-3, 33850310 PMC8093260

[16] Hu Y Wang B Li S Yang S. Pyroptosis, and its role in central nervous system disease. J Mol Biol. (2022) 434:167379. doi: 10.1016/j.jmb.2021.167379, 34838808

[17] Bellut M Bieber M Kraft P Weber ANR Stoll G Schuhmann MK. Delayed NLRP3 inflammasome inhibition ameliorates subacute stroke progression in mice. J Neuroinflammation. (2023) 20:4. doi: 10.1186/s12974-022-02674-w, 36600259 PMC9811791

[18] Ward R Li W Abdul Y Jackson LD Dong G Jamil S . NLRP3 inflammasome inhibition with MCC950 improves diabetes-mediated cognitive impairment and vasoneuronal remodeling after ischemia. Pharmacol Res. (2019) 142:237–50. doi: 10.1016/j.phrs.2019.01.035, 30818045 PMC6486792

[19] Han D Wang J Wen L Sun M Liu H Gao Y. Vinpocetine attenuates ischemic stroke through inhibiting NLRP3 inflammasome expression in mice. J Cardiovasc Pharmacol. (2020) 77:208–16. doi: 10.1097/FJC.0000000000000945, 33351536 PMC7853762

[20] Chen LG Jan YS Tsai PW Norimoto H Michihara S Murayama C . Anti-inflammatory and antinociceptive constituents of *Atractylodes japonica* Koidzumi. J Agric Food Chem. (2016) 64:2254–62. doi: 10.1021/acs.jafc.5b05841, 26919689

[21] Liu F Wang Y Li D Yang T. Atractylodin ameliorates lipopolysaccharide-induced depressive-like behaviours in mice through reducing neuroinflammation and neuronal damage. J Neuroimmunol. (2024) 390:578349. doi: 10.1016/j.jneuroim.2024.578349 38669915

[22] Yang L Ji C Li Y Hu F Zhang F Zhang H . Natural potent NAAA inhibitor atractylodin counteracts LPS-induced microglial activation. Front Pharmacol. (2020) 11:577319. doi: 10.3389/fphar.2020.577319, 33117168 PMC7565389

[23] Tang F Fan K Wang K Bian C. Atractylodin attenuates lipopolysaccharide-induced acute lung injury in association with reduced NLRP3 inflammasome activation and TLR4 pathways. J Pharmacol Sci. (2018) 136:203–11. doi: 10.1016/j.jphs.2017.11.010, 29551284

[24] Ma Y Wang J Wang Y Yang G-Y. The biphasic function of microglia in ischemic stroke. Prog Neurobiol. (2017) 157:247–72. doi: 10.1016/j.pneurobio.2016.01.005, 26851161

[25] Longa EZ Weinstein PR Carlson S Cummins R. Reversible middle cerebral artery occlusion without craniectomy in rats. Stroke. (1989) 20:84–91. doi: 10.1161/01.str.20.1.84, 2643202

[26] Shi Y-H Zhang X-L Ying P-J Wu Z-Q Lin L-L Chen W . Neuroprotective effect of Astragaloside IV on cerebral ischemia/reperfusion injury rats through Sirt1/Mapt pathway. Front Pharmacol. (2021) 12:639898. doi: 10.3389/fphar.2021.639898, 33841157 PMC8033022

[27] Alsbrook DL di Napoli M Bhatia K Biller J Andalib S Hinduja A . Neuroinflammation in acute ischemic and hemorrhagic stroke. Curr Neurol Neurosci Rep. (2023) 23:407–31. doi: 10.1007/s11910-023-01282-2, 37395873 PMC10544736

[28] Shi K Xiao Y Qu M Xie Y Wang Y Ke C . Atractylodin modulates ASAH3L to improve galactose metabolism and inflammation to alleviate acute lung injury. iScience. (2024) 27:110751. doi: 10.1016/j.isci.2024.110751, 39351199 PMC11440247

[29] Chen L Tang Y-L Liu Z-H Pan Y Jiao R-Q Kong L-D. Atractylodin inhibits fructose-induced human podocyte hypermotility via anti-oxidant to down-regulate TRPC6/p-CaMK4 signalling. Eur J Pharmacol. (2021) 913:174616. doi: 10.1016/j.ejphar.2021.174616 34780752

[30] Li Z Song Y Hou W Qi Y Lu X Xue Y . Atractylodin induces oxidative stress-mediated apoptosis and autophagy in human breast cancer MCF-7 cells through inhibition of the P13K/Akt/mTOR pathway. J Biochem Mol Toxicol. (2022) 36:e23081. doi: 10.1002/jbt.23081, 35478473

[31] Chen Y Ye X Escames G Lei W Zhang X Li M . The NLRP3 inflammasome: contributions to inflammation-related diseases. Cell Mol Biol Lett. (2023) 28:51. doi: 10.1186/s11658-023-00462-9, 37370025 PMC10303833

[32] Zhang J Liu X Wan C Liu Y Wang Y Meng C . NLRP3 inflammasome mediates M1 macrophage polarization and IL‐1β production in inflammatory root resorption. J Clin Periodontol. (2020) 47:451–60. doi: 10.1111/jcpe.13258, 31976565

