# Supplementary figures and images for: Atractylodin alleviates neuroinflammation and protects neurological function after ischemic stroke in association with reduced NLRP3 inflammasome activation
Source: Front Neurol. 2026 Mar 27;17:1754614. doi: 10.3389/fneur.2026.1754614 (PMC13065679; doi:10.3389/fneur.2026.1754614)

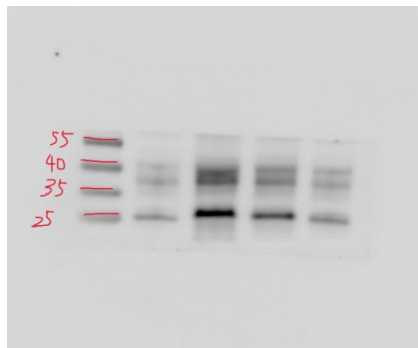

ASC

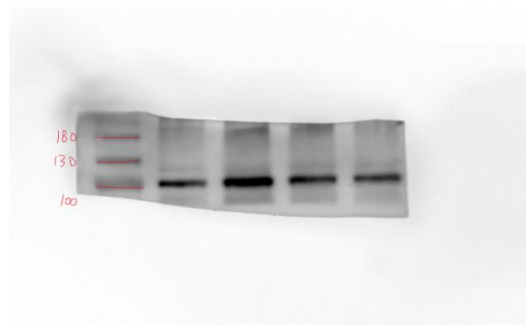

NLRP3

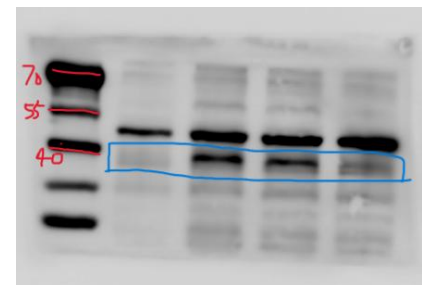

Caspase-1

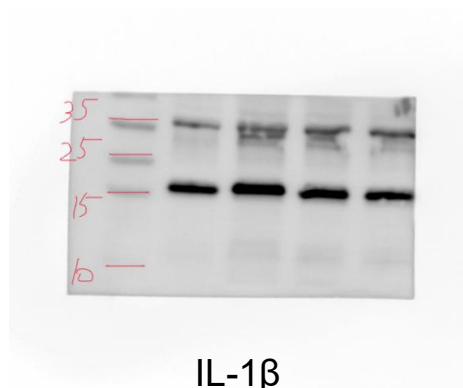

IL-1 $\beta$

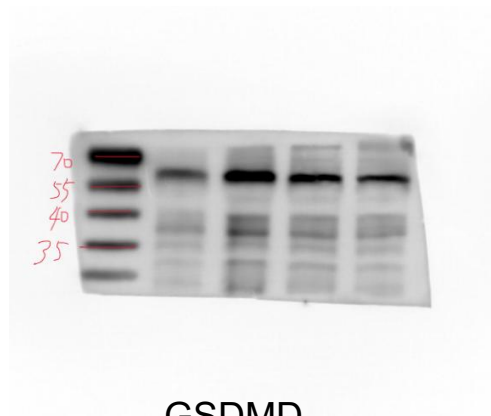

GSDMD

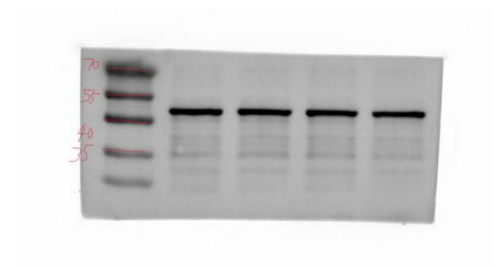

$\beta$ -actin

**supplement figure**

Supplement: Supplementary file 1 [file Data_Sheet_1.PDF]

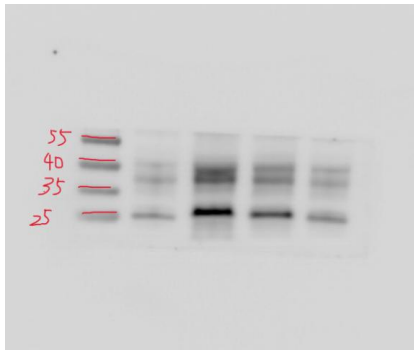

ASC

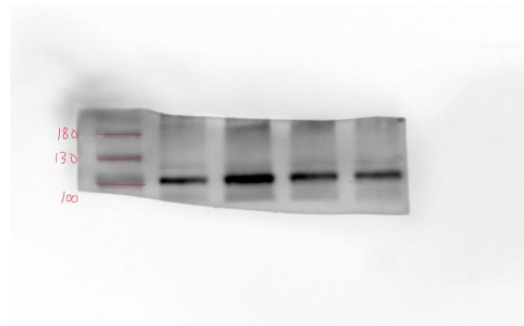

NLRP3

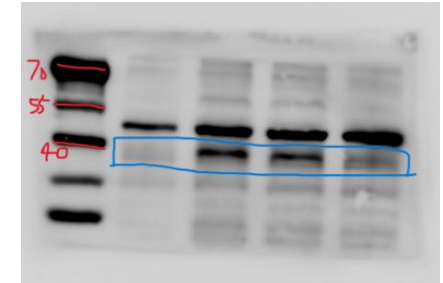

Caspase-1

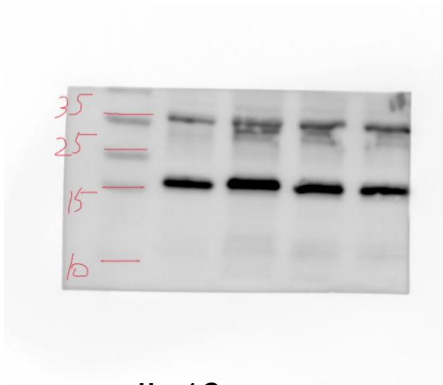

IL-1 $\beta$

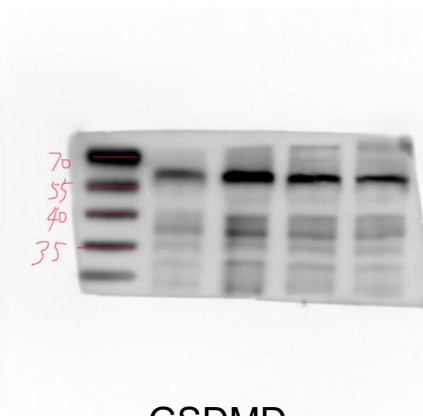

GSDMD

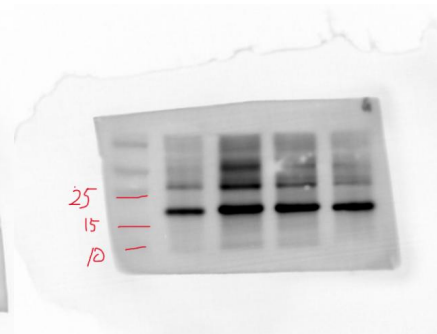

IL-18

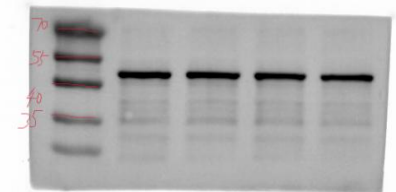

$\beta$ -actin

**supplement figure**

Supplement: Supplementary file 2 [file Data_Sheet_2.PDF]
